# Risk factors for totally implantable access ports-associated thrombosis in pediatric oncology patients

**DOI:** 10.1038/s41598-023-30763-0

**Published:** 2023-03-02

**Authors:** Yingxia Lan, Liuhong Wu, Jin Guo, Juan Wang, Huijie Guan, Baihui Li, Longzhen Liu, Lian Zhang, Ye Hong, Jun Deng, Jia Zhu, Suying Lu, Feifei Sun, Junting Huang, Xiaofei Sun, Yizhuo Zhang, Jian Wang, Ruiqing Cai

**Affiliations:** 1 Sun Yat-Sen University Cancer Center, State Key Laboratory of Oncology in South China, Collaborative Innovation Center for Cancer Medicine, Guangzhou, 510060 Guangdong People’s Republic of China; 2 Department of Pediatric Oncology, Sun Yat-Sen University Cancer Center, Guangzhou, 510060 Guangdong People’s Republic of China; 3 Department of Anesthesiology and Operating Theatre, Sun Yat-Sen University Cancer Center, Guangzhou, 510060 Guangdong People’s Republic of China

**Keywords:** Paediatric cancer, Oncology, Risk factors

## Abstract

The application of totally implantable access ports (TIAPs) reduces treatment-related discomfort; however, the existence of catheter may cause side effects, with the most common one being the occurrence of TIAPs-associated thrombosis. The risk factors for TIAPs-associated thrombosis in pediatric oncology patients have not been fully described. A total of 587 pediatric oncology patients undergoing TIAPs implantation at a single center over a 5-year period were retrospectively analyzed in the present study. We investigated the risk factors for thrombosis, emphasizing the internal jugular vein distance, by measuring the vertical distance from the highest point of the catheter to the upper border of the left and right extremitas sternalis claviculae on chest X-ray images. Among 587 patients, 143 (24.4%) had thrombosis. Platelet count, C-reactive protein, and the vertical distance from the highest point of the catheter to the upper border of the left and right extremitas sternalis claviculae were demonstrated to be the main risk factors for the development of TIAPs-associated thrombosis. TIAPs-associated thrombosis, especially asymptomatic events, is common in pediatric cancer patients. The vertical distance from the highest point of the catheter to the upper border of the left and right extremitas sternalis claviculae was a risk factor for TIAPs-associated thrombosis, which deserved additional attention.

## Introduction

Cancer patients are prone to thromboembolism. Thrombosis results from coagulation dysfunction, which is caused by various factors such as individual patient factors, disease status, and treatment regimens^1^. Among these factors, previous studies have identified central venous catheter (CVC) as an independent factor associated with an increased risk of VTE in cancer patients^2^. In recent years, the use of TIAPs in cancer patients has gradually increased due to its long indwelling time, long maintenance period, and improved quality of life over other CVCs^3,4^. TIAP has become especially popular among pediatric oncology patients, as it can reduce unintentional pullout of exposed catheters commonly occurring in children and is more likely to produce positive body images than other CVCs.

The currently reported incidence of catheter-related thrombosis (CRT) varies widely, depending on symptomatic events or whether patients are screened for asymptomatic thrombosis^5^. CRT incidence in pediatric cancer population may differ from that in adults^6^. And in a large study^7^, risk factors for CRT differed from those for noncatheter-related venous thromboembolism. Although some studies have analyzed the risk factors for CVC-related thrombosis^8,9^, most of them adopt peripherally inserted central catheter (PICC), and the risk factors for TIAPs-associated thrombosis have not been fully described. Therefore, the aim of the present study was to elucidate the risk factors for TIAPs-associated thrombosis in pediatric oncology patients.

## Materials and methods

### Study population

A total of 587 pediatric oncology patients who underwent TIAPs implantation at Sun Yat-sen University cancer center between January 2016 and October 2020 were retrospectively included in our study. Only newly diagnosed with malignancy children younger than 18 years at the time of port insertion were included. Patients who lost follow-up after initial examination and treatment were excluded. TIAPs placement was performed by a professional surgeon and anesthesia team, and all port implantation was guided by Doppler ultrasound. Catheter depth is determined by anatomical landmark technique. Each patient underwent a chest X-ray after port insertion to detect possible misplacements and to rule out pleura-pulmonary complications such as pneumothorax or hemothorax. As for the choice of catheter, we keep the outer diameter of the catheter no more than 1/3 of the internal diameter of the vein. Blood routine examination, coagulation function, and infectious disease screening were completed for each patient before port placement. Patients’ data were followed up by telephone and access to outpatient and inpatient data. The endpoint was the port removal or the last day of follow-up (December 31, 2021). Our study was approved by the Ethic Committee of Sun Yat-sen University Cancer Center (Approval Number: B2022-197-01) and conducted in accordance with the latest version of the Declaration of Helsinki. The written informed consent was obtained from all participants and/or their legal guardians.

### Analysis of TIAPs-associated thrombosis

The TIAPs-associated thrombosis was confirmed by ultrasound imaging, and no radiographic imaging was used due to its toxicity in children. We explored the clinical presentations of thrombosis, including insertion location and outcome. In addition, the association of thrombosis with age, gender, body mass index (BMI), tumor type, disease stage, albumin, C-reactive protein (CRP), white blood cell (WBC) count, hemoglobin level, platelet count, d-Dimer, international normalized ratio (INR), activated partial prothrombin time (APTT), and the vertical distance from the highest point of the catheter to the upper border of the left and right extremitas sternalis claviculae in chest X-ray images were analyzed.

### Statistical analysis

All analyses were done by IBM SPSS Statistics version 19.0 (SPSS, Chicago, IL, USA) software. The Chi-square test was used for comparisons of qualitative variables between the groups, if appropriate. Continuous variables were compared using the Student’s test, as appropriate. All variables that affected thrombosis (level of significance, P < 0.1) in the univariate analysis were included in the multivariate logistic regression analysis. A P-value of < 0.05 was considered statistically significant.

## Results

### Patient characteristic

Over the 5-year study period, 587 consecutive newly diagnosed pediatric oncology patients were included. The patient characteristics are summarized in Table 1. Among these patients, 143 (24.4%) developed TIAPs-associated thrombosis. The median age was 4.0 (range 0.1–16) years, and the male to female ratio was 1.4. For patients with thrombosis, the duration of port insertion was defined as the time from TIAPs placement to port removal or the last follow-up time, with an average of 29.5 (range 1.3–72.4) months. At the end of follow-up, 39.2% (56/143) of patients with thrombosis had completed treatment and the port had been removed. The mean time from thrombus onset to port insertion was 24.3 days, with a median of 18.0 (range 3–488) days. The median interval between the first ultrasound imaging for thrombosis was 13.0 (range 3–1209) days. Also, the median interval between the second ultrasound imaging was 21 (range 4–890) days.

**Table 1:** Patient characteristics (n = 587).

Characteristics	VTE (+) n (%)	VTE (−) n (%)	Total n (%)	*P*-value
Gender				0.277
Male	77 (53.8)	262 (59.0)	339 (57.8)	
Female	66 (46.2)	182 (41.0)	248 (42.2)	
Age (years)				0.945
Mean (min–max)	4.7 (0.1–16)	4.7 (0.3–13)	4.7	
Median	4.0	4.0	4.0	
BMI				0.426
Mean (min–max)	15.6 (10.2–25.6)	15.6 (10.1–27.6)	15.6	
Median	15.3	15.3	15.3	
Stage				0.969
Early	39 (27.3)	117 (26.4)	156 (26.6)	
Advanced	98 (68.5)	307 (69.1)	405 (69.0)	
Recurrence	6 (4.2)	20 (4.5)	26 (4.4)	
WBC (× 10^9^/L)				0.617
Mean (min–max)	8.6 (1.28–23.01)	8.4 (0.79–63.09)	8.4	
Median	8.14	7.4	7.6	
Hemoglobin (g/L)				0.073
Mean (min–max)	114.3 (55–155)	110.7 (28–158)	111.6	
Median	118.0	115.0	115.0	
Platelet count (× 10^9^/L)				0.001
Mean (min–max)	381.3 (37–1156)	336.9 (9–1020)	347.7	
Median	377.0	322.0	332.5	
Albumin (g/L)				0.745
Mean (min–max)	43.5 (30.2–114.8)	43.7 (23.2–197.1)	43.6	
Median	43.5	43.3	43.3	
d-Dimer (mg/L)				0.411
Mean (min–max)	8.5 (0.09–121.19)	10.1 (0.05–129.09)	9.7	
Median	0.9	1.2	1.1	
APTT (s)				0.469
Mean(min–max)	24.4 (0.1–42.2)	25.4 (0.12–121)	25.1	
Median	27.3	27.8	27.6	
INR				0.239
Mean (min–max)	1.01 (0.79–1.31)	1.06 (0.82–1.77)	1.06	
Median	1.02	1.04	1.03	
CRP (mg/L)				0.002
Mean (min–max)	8.2 (0.03–88.12)	14.9 (0.02–300.9)	13.3	
Median	1.2	1.6	1.5	
Distance^a^ (mm)				0.05
Mean (min–max)	17.1 (7–41)	15.8 (5–58)	16.1	
Median	15.5	15.0	15.0	
Drug^b^				0.273
a	106 (74.1)	353 (79.5)	459 (78.2)	
b	18 (12.6)	37 (8.3)	55 (9.4)	
c	19 (13.3)	54 (12.2)	73 (12.4)	

^a^Distance defines the vertical distance from the highest point of the catheter to the upper border of the left and right extremitas sternalis claviculae.

^b^a: Not use dexamethasone and asparaginase, b: Use dexamethasone and asparaginase, c: Only use dexamethasone.

**Figure 1:**
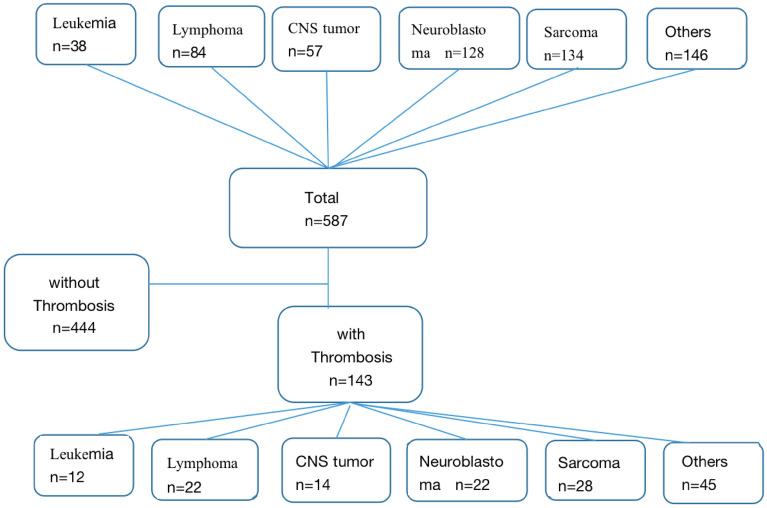
Flow chart of study populations.

We classified all pediatric oncology patients into five major groups: leukemia, lymphoma, neuroblastoma, sarcoma, and other types of tumor (Fig. 1). In terms of tumor types, leukemia and other types had a higher incidence of thrombosis, while neuroblastoma and sarcoma had a lower incidence. Leukemia was statistically significant compared with neuroblastoma (Odds ratio [OR] 2.31, P = 0.043), while other types were more prone to thrombosis than neuroblastoma (OR 2.17, P = 0.008) and sarcoma (OR 1.72, P = 0.05).

### Site features of TIAPs-associated thrombosis

Among 143 patients with thrombosis (Table 2), in 67.8% (97/143) patients, it occurred in the right internal jugular vein. In 26 patients with thrombosis in the right subclavian vein, the port insertion was the right internal jugular vein. Two patients with thrombosis in the right brachiocephalic vein also had an insertion vessel in the right internal jugular vein, and one patient with thrombosis in the left subclavian vein had a port insertion site in the left internal jugular vein. The internal jugular vein was more prone to thrombosis than the subclavian vein; yet, the difference was not statistically significant (OR 1.56, P = 0.163).

**Table 2:** Sites of TIAPs-associated thrombosis in pediatric oncology patients.

	Insertion position of thrombosis patients	Site of thrombus	Insertion position of all patients
Right internal jugular, n (%)	125 (87.4)	97 (67.8)	488 (83.1)
Right subclavian, n (%)	12 (8.4)	38 (26.6)	70 (11.9)
Left internal jugular, n (%)	6 (4.2)	5 (3.5)	26 (4.4)
Left subclavian, n (%)	0 (0)	1 (0.7)	3 (0.5)
Right brachipcephalic vein, n (%)	0 (0)	2 (1.4)	0 (0)

### Risk factors for developing TIAPs-associated thrombosis

Hemoglobin (P = 0.073), platelet count (P = 0.001), CRP (P = 0.002), and the vertical distance from the highest point of the catheter to the upper border of the left and right extremitas sternalis claviculae (P = 0.05) were identified as risk factors for developing TIAPs-associated thrombosis. Multivariate analysis revealed that platelet count, CRP, and the vertical distance from the highest point of the catheter to the upper border of the left and right extremitas sternalis claviculae remained significant risk factors (Table 3).

**Table 3:** Risk factors by multivariate logistic regression analysis.

	Odds ratio	95% CI (lower–upper)	P-value
Hemoglobin	1.004	0.993–1.016	0.481
Platelet count	1.002	1.001–1.003	0.004
CRP	0.990	0.980–1.000	0.054
Distance	1.031	1.001–1.062	0.044

## Discussion

CVC is a well-known risk factor for thrombosis; however, only a few studies have investigated the risk factors for thrombosis associated with TIAP use in pediatric cancer patients^10,11^. Our study focused on risk factors for TIAPs-associated thrombosis, especially the vertical distance from the highest point of the catheter to the upper border of the left and right extremitas sternalis claviculae. This has not been reported in previous studies and could help early identification of populations at high risk of thrombosis. Identification of risk factors for CRT in childhood cancer patients before port implantation may greatly contribute to prognosis improvement.

In a prospective multicenter French cohort study (ONCOCIP)^7^, the only independent risk factor for catheter-related thrombosis in adult cancer patients with port implants was the use of cephalic vein for catheter insertion. Specific factors may be related to the occurrence of TIAPs-associated thrombosis. Since previous studies have shown that right internal jugular vein insertion is linked with the lowest incidence of thrombosis^12,13^, 83.1% (488/587) of patients in our center have chosen right internal jugular vein insertion. The remaining patients were unable to choose that site mostly due to the influence of diseases such as right mediastinal mass. Therefore, our findings suggested a higher incidence of thrombosis in the right internal jugular vein insertion, probably because most children chose this site.

Previous studies have shown that the use of dexamethasone and asparaginase are risk factors for thrombosis^14^; yet, we did not observe similar findings, which might be related to the fact that our pediatric patients had all types of childhood tumors and received different treatment regimens and medication. While cancer patients are prone to thrombosis, especially for those with hematological tumors such as leukemia^15^. Some cancer types are associated with a lower incidence of thrombosis, such as central nervous system (CNS) tumors that have been previously reported^16^. In our study, leukemia was significantly prone to thrombosis; neuroblastoma and sarcoma had the lowest thrombosis incidence of 17.2% (22/128) and 20.9% (28/134), respectively, which were similar to previous findings^10^.

In previous studies^4^, many CRT risk factors were discovered, including PICC instead of port, but our study only included TIAP patients. In a chemotherapy-related VTE model^17^, a platelet count > 350 × 10^9^/L was one of the five predictors for VTE. The Compass-CAT risk prediction model^18^ still included a platelet count > 350 × 10^9^/L as a risk factor. Some studies^7^ have also shown that antiplatelet therapy has a protective effect against CRT. Similar to other studies, platelet count was found to be a risk factor for CRT in our study. Current evidence is conflicting as to whether inflammation levels or CRP are risk factors for VTE^19^. Studies of CRP in cancer populations have not focused on CRT^20,21^. In a multicenter prospective study^22^, CRP levels were found to predict venous thromboembolism recurrence after discontinuation of anticoagulation for cancer-associated thrombosis. Overweight or obesity is a prothrombotic state in which risk factors for venous thrombosis are inflammatory responses, decreased fibrinolysis, increased thrombin production, and platelet hyperactivity^23^. In our study, few children were obese, but CRP was still a risk factor for CRT, which might be related to the different characteristics of catheter-related thrombosis versus non-catheter-related thrombosis. It was also possible that our patients all had malignant tumors, which were risk factors for thrombosis, and that thrombosis could be regulated by inflammatory markers^24^.

Due to slow blood flow velocity of the internal jugular vein and contralateral compensation, the blood flow velocity will be further reduced after port insertion, so patients with long internal jugular vein catheter may be prone to thrombosis. At present, the accurate judgment of the distance of the internal jugular vein needs to be determined by computed tomograph (CT). In order to reduce radiation doses, we generally chose chest X-ray to determine the correct placement of the catheter after port implantation. In addition, because of the error in the measurement of the distance from the puncture point to the extremitas sternalis claviculae during the operation, the vertical distance from the highest point of the catheter to the upper border of the left and right extremitas sternalis claviculae in radiographic images was regarded as the distance of the internal jugular vein. We found it to be one of the risk factors for TIAPs-associated thrombosis by univariate and multivariate analysis. The distance was longer in patients with thrombosis, averaging 17.1 (range 7–41) mm, compared with 15.8 (range 5–58) mm in patients without thrombosis. Our study identified a new risk factor for port patients with internal jugular vein insertion that was easily observed. Therefore, we recommended the puncture site as low as possible to reduce the length of the internal jugular vein catheter. However, because low site puncture could easily penetrate the subclavian artery, blind puncture was not advisable. Doppler ultrasound-guided puncture would be recommended. However, since this was a retrospective study, more large clinical trials were needed to further verify our results.

In our center, in order to detect thrombus in time, ultrasound imaging of the upper extremity was routinely performed on the 7th and 21st days after port insertion. Consistent with previous reports^25^, most thrombotic events in our study occurred during the first 3 months of follow-up. Our data showed that in 28.7% (41/143) patients, thrombus was found on the 7th day of routine ultrasound imaging; in 22.4% (32/143) patients, it was found on the 21st day, and in the remaining patients, some was found after the appearance of symptoms. Nevertheless, since this was a retrospective study, the time of the first ultrasound imaging was not available for some patients because the examination was performed in other hospitals, and the report could not be obtained. At present, there are no guidelines on when to perform an examination after TIAP placement. PICC-related thrombosis is reported to be a common and almost always asymptomatic complication in children^26^. Children with CVC-associated thrombosis often have recurrent catheter-related complications. Therefore, asymptomatic thrombosis may be clinically important. In a prospective study^27^, the incidence of asymptomatic DVT and fibrin sheath monitored by Doppler ultrasound at 1-, 6-, and 12-month after implantation in cancer patients with long-term CVC implantation was 0.10 events per 1000 catheter days (< 1.5%). Therefore, asymptomatic patients were more likely to be detected using early ultrasound. Our study found 24.4% of patients with thrombosis, most of whom were asymptomatic, and half of them were detected on day 7 and 21 after TIAP implantation. At our center, which is a large tumor research center in China, we accumulated a lot of experience in the management of thrombus in tumor patients, which we could recommend for thrombus screening in the future.

This study had some limitations. On the one hand, this was a retrospective study with patient selection bias. On the other hand, our patients had all types of childhood tumors and received different treatment regimens and medication, so it is difficult to determine the drug-related effect on thrombosis.

In conclusion, platelet count, CRP, and the vertical distance from the highest point of the catheter to the upper border of the left and right extremitas sternalis claviculae might be the risk factors for TIAPs-associated thrombosis. We recommend ultrasound imaging on day 7 and 21 after port insertion in pediatric oncology patients for early detection of asymptomatic thrombotic events. Moreover, the puncture position should be as low as possible to reduce the length of catheter in the internal jugular vein, which might reduce the occurrence of TIAPs-associated thrombosis.

## Data Availability

The datasets used and/or analysed during the current study available from the corresponding author on reasonable request.

## Abbreviations

TIAPs: Totally implantable access ports
CRP: C-reactive protein
CVC: Central venous catheter
CRT: Catheter-related thrombosis

## References

[1] Fernandes CJ, Morinaga LTK, Alves JL (2019). Cancer-associated thrombosis: The when, how and why. Eur. Respir. Rev..

[2] Ashrani AA, Gullerud RE, Petterson TM (2016). Risk factors for incident venous thromboembolism in active cancer patients: A population based case-control study. Thromb. Res..

[3] Ignatov A, Hoffman O, Smith B (2009). An 11-year retrospective study of totally implanted central venous access ports: Complications and patient satisfaction. Eur. J. Surg. Oncol..

[4] Fang S, Yang J, Song L, Jiang Y, Liu Y (2017). Comparison of three types of central venous catheters in patients with malignant tumor receiving chemotherapy. Patient Prefer. Adherence..

[5] Marin A, Bull L, Kinzie M, Andresen M (2021). Central catheter-associated deep vein thrombosis in cancer: Clinical course, prophylaxis, treatment. BMJ Support Palliat. Care..

[6] Albisetti M, Kellenberger CJ, Bergsträsser E (2013). Port-a-cath-related thrombosis and postthrombotic syndrome in pediatric oncology patients. J. Pediatr..

[7] Decousus H, Bourmaud A, Fournel P (2018). Cancer-associated thrombosis in patients with implanted ports: A prospective multicenter French cohort study (ONCOCIP). Blood.

[8] Tabatabaie O, Kasumova GG, Kent TS (2017). Upper extremity deep venous thrombosis after port insertion: What are the risk factors?. Surgery..

[9] Revel-Vilk S, Yacobovich J, Tamary H (2010). Risk factors for central venous catheter thrombotic complications in children and adolescents with cancer. Cancer.

[10] Wiegering V, Schmid S, Andres O (2014). Thrombosis as a complication of central venous access in pediatric patients with malignancies: A 5-year single-center experience. BMC Hematol..

[11] Citla Sridhar D, Abou-Ismail MY, Ahuja SP (2020). Central venous catheter-related thrombosis in children and adults. Thromb. Res..

[12] Verso M, Agnelli G, Kamphuisen PW (2008). Risk factors for upper limb deep vein thrombosis associated with the use of central vein catheter in cancer patients. Intern. Emerg. Med..

[13] Debourdeau P, Farge D, Beckers M (2013). International clinical practice guidelines for the treatment and prophylaxis of thrombosis associated with central venous catheters in patients with cancer. J. Thromb. Haemost..

[14] Chen K, Agarwal A, Tassone MC (2016). Risk factors for central venous catheter-related thrombosis in children: A retrospective analysis. Blood Coagul Fibrinolysis..

[15] Kekre N, Connors JM (2019). Venous thromboembolism incidence in hematologic malignancies. Blood Rev..

[16] Howie C, Erker C, Crooks B, Moorehead P, Kulkarni K (2021). Incidence and risk factors of venous thrombotic events in pediatric patients with CNS tumors compared with non-CNS cancer: A population-based cohort study. Thromb. Res..

[17] Khorana AA, Kuderer NM, Culakova E, Lyman GH, Francis CW (2008). Development and validation of a predictive model for chemotherapy-associated thrombosis. Blood.

[18] Gerotziafas GT, Taher A, Abdel-Razeq H (2017). A predictive score for thrombosis associated with breast, colorectal, lung, or ovarian cancer: The prospective COMPASS-cancer-associated thrombosis study. Oncologist..

[19] Galeano-Valle F, Ordieres-Ortega L, Oblitas CM (2021). Inflammatory biomarkers in the short-term prognosis of venous thromboembolism: A narrative review. Int. J. Mol. Sci..

[20] Kanz R, Vukovich T, Vormittag R (2011). Thrombosis risk and survival in cancer patients with elevated C-reactive protein. J. Thromb. Haemost..

[21] Kröger K, Weiland D, Ose C (2006). Risk factors for venous thromboembolic events in cancer patients. Ann. Oncol..

[22] Jara-Palomares L, Solier-Lopez A, Elias-Hernandez T (2018). D-dimer and high-sensitivity C-reactive protein levels to predict venous thromboembolism recurrence after discontinuation of anticoagulation for cancer-associated thrombosis. Br. J. Cancer..

[23] Samad F, Ruf W (2013). Inflammation, obesity, and thrombosis. Blood.

[24] Saghazadeh A, Hafizi S, Rezaei N (2015). Inflammation in venous thromboembolism: Cause or consequence?. Int. Immunopharmacol..

[25] Hohl Moinat C, Périard D, Grueber A (2014). Predictors of venous thromboembolic events associated with central venous port insertion in cancer patients. J. Oncol..

[26] Menéndez JJ, Verdú C, Calderón B (2016). Incidence and risk factors of superficial and deep vein thrombosis associated with peripherally inserted central catheters in children. J. Thromb. Haemost..

[27] Boddi M, Villa G, Chiostri M (2015). Incidence of ultrasound-detected asymptomatic long-term central vein catheter-related thrombosis and fibrin sheath in cancer patients. Eur. J. Haematol..

